# Late-onset cluster seizures and intellectual disability associated with a novel truncation variant in *SMC1A*

**DOI:** 10.1016/j.ebr.2022.100556

**Published:** 2022-06-02

**Authors:** Menatalla Elwan, Ross Fowkes, David Lewis-Smith, Amy Winder, Mark R. Baker, Rhys H. Thomas

**Affiliations:** a Department of Neurology, Royal Victoria Infirmary, Newcastle upon Tyne NE1 4LP, United Kingdom; b Translational and Clinical Research Institute, Newcastle University, Newcastle upon Tyne NE2 4HH, United Kingdom; c Department of Clinical Neurophysiology, Royal Victoria Infirmary, Newcastle upon Tyne NE1 4LP, United Kingdom; d Wellcome Centre for Mitochondrial Research, Newcastle University, Newcastle upon Tyne NE2 4HH, United Kingdom

**Keywords:** *SMC1A*, Epilepsy, *PCDH19*, Clustering seizures

## Abstract

*SMC1A* variants are known to cause Cornelia de Lange Syndrome (CdLS) which encompasses a clinical spectrum of intellectual disability, dysmorphic features (long or thick eyebrows, a hypomorphic philtrum and small nose) and, in some cases, epilepsy. More recently, *SMC1A* truncating variants have been described as the cause of a neurodevelopmental disorder with early-childhood onset drug-resistant epilepsy with seizures that occur in clusters, similar to that seen in *PCDH19*-related epilepsy, but without the classical features of CdLS. Here, we report the case of a 28-year-old woman with a *de novo* heterozygous truncating variant in *SMC1A* who unusually presented with seizures at the late age of 12 years and had normal development into adulthood.

## Highlights

• Epilepsy due to truncating *SMC1A* variants can present with onset in later childhood.
• People with to truncating *SMC1A* variants can have normal development prior to presentation.
• Seizures occur periodically in clusters and are poorly responsive to antiseizure medications.

## 1 Introduction

1.1 Genetic diagnosis and counselling for the monogenic epilepsies is critical because not all are caused by *de novo* dominant variants. An important example is the epilepsy associated with *PCDH19,* on the X chromosome. *PCHD19* variants cause an epilepsy with clusters of focal-onset and fever-sensitive seizures, primarily restricted to females and within a spectrum of cognitive impairment and psychiatric comorbidity [1], [2]. This is phenotypically similar to the presentation of women with pathogenic variants in another X chromosome gene, *SMC1A* [3].

1.2 The *SMC1A* gene, Xp11.22, encodes a subunit of the cohesin complex. This complex has several functions including the holding together of sister chromatids, thereby ensuring chromosome segregation during cell replications, modulation of gene expression, and DNA repair [4]. *De novo* variants in *SMC1A* were first known to be a rare cause of Cornelia de Lange Syndrome (CdLS); (4–6 % of CdLS patients). CdLS encompasses a spectrum of clinical features characterized predominantly by intellectual disability, facial dysmorphism, growth restriction, hypertrichosis, and congenital defects of the upper limbs, gastrointestinal tract, heart, and genitourinary tract [5]. Epilepsy is present in approximately 20% of people with CdLS, typically with focal-onset seizures which are relatively easily controlled with antiseizure medications [6]. CdLS attributed to variants in *SMC1A* generally manifests a milder or less classical phenotype than when attributed to variants in the most common cause, *NIPBL* [7].

More recently, protein truncating variants in *SMC1A* have been reported in association with a developmental and epileptic encephalopathy that can occur with or without midline brain defects (DEE85, OMIM: 301044). These cases are associated with a distinct phenotype of neurodevelopmental disorders and drug-resistant epilepsy but without the typical features of CdLS, or with features suggestive of Rett syndrome [3], [8], [9], [7], [10], [11], [12], [13]. These include a case series of 10 female people with moderate to severe developmental impairment and drug-resistant seizures, which showed a clustering pattern [3]. In all these previously reported cases with epilepsy, seizures presented in early childhood and usually in infancy.

1.3 We report the case of a 28-year-old woman with drug-resistant epilepsy and normal neurodevelopment who developed seizures late, at the age of 12 years, and who carries a novel *de novo* heterozygous truncating variant in *SMC1A*.

## 2 Case report

2.1 This 28-year-old woman first presented to the pediatric neurology service aged 12 years with unprovoked presumed generalized onset tonic-clonic seizures. She was the product of an uncomplicated pregnancy and spontaneous delivery at term. She had normal development prior to seizure onset, although was described as having clumsy gross motor skills and being poor at sports at the age of 12. There was no impairment of fine motor or language development. Examination identified bilateral pes cavus, hyporeflexia in the lower limbs, a broad-based gait, non-progressive peripheral neuropathy, and a mild degree of facial asymmetry.
2.2 Her seizures were initially characterized by clusters of presumed generalized tonic-clonic seizures in the context of a febrile illness. She commenced phenytoin, supplemented by topiramate without control of seizures. She also developed focal seizures characterized by impaired awareness and right upper limb motor spasms, as well as occasional generalized myoclonic seizures. By 16 years of age, she developed monthly clusters of seizures, with a catamenial tendency, resulting in multiple admissions to critical care due to convulsive status epilepticus. At this point her antiseizure medication was changed to levetiracetam and phenytoin, due to a metabolic acidosis caused by topiramate, and she commenced levonorgestrel/ethinylestradiol to halt ovulation in the hope that this would aid seizure control. This coincided with 18 months of seizure freedom, during which she was able to start undergraduate studies in college, which she completed with a passing grade at the age of 21.
  At the age of 20, she developed focal autonomic seizures. These were stereotyped episodes of flushing, followed by deep inspiration, increasingly loud and repetitive speech, and an appearance of being vacant. These occurred in addition to clusters of generalised tonic-clonic seizures of unknown onset occurring five to eight times per day. Clobazam and lamotrigine were introduced in addition to levetiracetam and phenytoin. By this time, her cognitive function measured in an interictally had declined, and an Addenbrooke’s Cognitive Examination – Revised (ACE-R) was 79/100 [14].
She continued to present to hospital monthly with predominantly convulsive, but also episodes of non-convulsive, status epilepticus. Her medications were changed to phenobarbital, phenytoin, and lacosamide, in addition to buccal midazolam and rectal paraldehyde in the event of status epilepticus. At the age of 28, she underwent a neuropsychological assessment which included assessments of premorbid intellectual function and current intellectual ability (assessed using Wechsler adult intelligence scale- IV in an interictal period [15]). Her IQ was estimated to be 68 (2nd percentile) and there was particular difficulty noted with delayed recall, attention control, executive function, and verbal and category fluency. Her current therapy consists of a vagus nerve stimulator, levetiracetam 750 mg b.d. and phenytoin 175 mg b.d. with seizure recurrence every 2 to 3 weeks. Cenobamate is currently being introduced. There does not appear to have been any clear relationship between anti-seizure medications and seizure frequency. See Table 1 for a summary of seizure frequency, medication, and adjunctive treatments to date.

**Table 1:** 

Year	Seizure Frequency	Episodes of Status Epilepticus	Anti-Seizure Medications	Adjunctive Therapies
2005	March – first and second seizure April – SE May – “frequent seizures” July – one generalized seizure	N Convulsive SE N N	Sodium valproate and carbamazepine – stopped due to Stevens-Johnson syndrome. Topiramate – stopped due to metabolic acidosis Levetiracetam	N
2006	Seizure-free	N	Levetiracetam	N
2007	Predominantly seizure-free	N	Levetiracetam	N
2008	Increased seizure frequency (monthly clusters) June – SE August Oct – SE	N Convulsive SE N Convulsive SE	Levetiracetam Levetiracetam Levetiracetam and phenytoin	Levonorgestrel/Ethinylestradiol
2009	Seizure-free		Levetiracetam and phenytoin	Levonorgestrel/Ethinylestradiol
2010	Seizure-free		Levetiracetam and phenytoin	Levonorgestrel/Ethinylestradiol
2011	Seizure-free		Levetiracetam and phenytoin	Levonorgestrel/Ethinylestradiol
2012	July – one generalized seizure	N	Levetiracetam and phenytoin	Levonorgestrel/Ethinylestradiol
2013	March – focal autonomic seizures July – SE Sept – monthly seizure clusters Oct – monthly seizure clusters Nov – monthly seizure clusters Dec – monthly seizure clusters	N Convulsive SE N N Convulsive SE N	Levetiracetam and phenytoin Levetiracetam and phenytoin Lamotrigine, levetiracetam and phenytoin Lamotrigine, levetiracetam and phenytoin Lamotrigine and phenytoin Lamotrigine, phenytoin, zonisamide	Levonorgestrel/Ethinylestradiol Medroxyprogesterone acetate
2014	Jan-July – monthly seizure clusters Aug – SE Sept-Dec – monthly seizure clusters	N Convulsive SE N	Phenytoin and zonisamide Feb – April. Perampanel added May. Perampanel and phenytoin Perampanel and phenytoin	Medroxyprogesterone acetate
2015	Jan-Dec – monthly seizure clusters	N	Levetiracetam and phenytoin	Medroxyprogesterone acetate Jan-April. Levonorgestrel/Ethinylestradiol May onwards.
2016	Jan-Sept – 8 admissions with seizure clusters Oct-Dec – 3 admissions with seizure clusters	Convulsive SE – Feb N	Levetiracetam and phenytoin Lacosamide, levetiracetam and phenytoin	Levonorgestrel/Ethinylestradiol
2017	Jan-May – 6 admissions. 1 with a single seizure, 1 with a seizure cluster, 4 with SE June-July – 4 admissions. 1 with a single seizure, 3 with SE. Aug-Sept – 5 admissions. 1 with a single seizure, 3 with seizure clusters, 1 with SE. Sept-Oct – 4 admissions. 1 with a seizure cluster, 3 with SE. Nov-Dec – 4 admissions. 2 with seizure clusters, 2 with SE.	Convulsive SE – Jan, March, April, May. Convulsive SE June, July. NCSE July. Convulsive SE Aug. Convulsive SE Oct. NCSE Sept, Oct. Convulsive SE Nov, Dec.	Lacosamide, levetiracetam and phenytoin Brivaracetam, lacosamide, phenytoin Lacosamide, levetiracetam and phenytoin Levatiracetam and phenytoin. Levetiracetam, phenobarbital, phenytoin.	Levonorgestrel/Ethinylestradiol Medroxyprogesterone acetate
2018	Jan-March – 4 admissions with seizure clusters. April-Dec – 19 admissions. 8 with singles seizures, 9 with seizure clusters, 2 with SE.	N Convulsive SE July and Dec.	Brivaracetam, phenytoin and phenobarbital. Single dose eslicarbazepine caused rash. Lacosamide, phenytoin and phenobarbital	Medroxyprogesterone acetate
2019	Jan – 1 admission with a single seizure. Feb-March – 2 admissions with seizure clusters. April-Dec – 15 admissions with seizure clusters.	N N N	Lacosamide and phenytoin Phenytoin Levetiracetam and phenytoin	Medroxyprogesterone acetate
2020	Jan – Dec – 21 admissions. 13 with single seizures, 4 with seizure clusters and 4 with SE.	NCSE Feb, July, Oct	Levetiracetam and phenytoin	Medroxyprogesterone acetate VNS June onwards
2021	Jan-July – 11 admissions. 5 with single seizures, 5 with seizure clusters, 1 with SE. Aug-Dec – 13 admissions. 8 with single seizures, 5 with seizure clusters.	Convulsive SE Jan N	Levetiracetam and phenytoin Cenobamate, levetiracetam and phenytoin	Medroxyprogesterone acetate VNS

SE (Status epilepticus), No (N), VNS (vagal nerve stimulator).

**Fig. 1:**
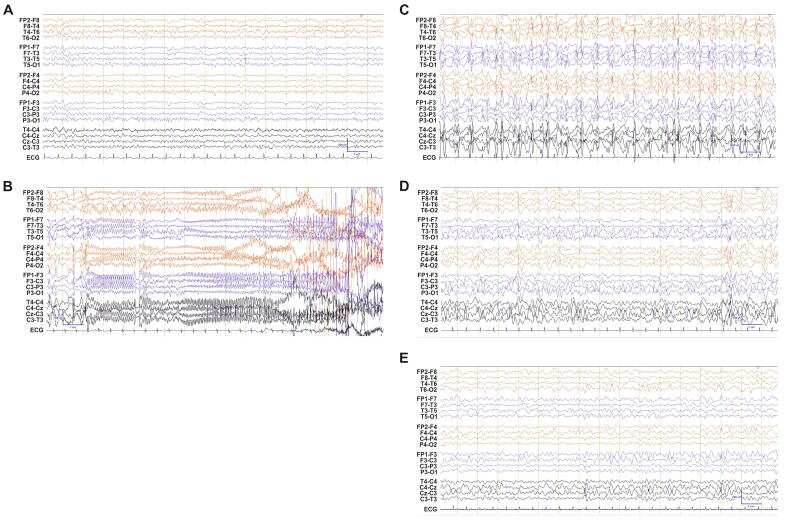
EEG recording. A. Typical EEG when well. Routine EEG performed before any episodes of status, age 20. Alpha rhythm present at 10–11 Hz. B. Generalized tonic-clonic seizure occurring during a period of *status epilepticus*. Patient unresponsive. Age 26. Build-up of generalized rhythmic 10 Hz activity with subsequent EMG and movement artefact. C. Nonconvulsive *status epilepticus*. Patient partially responsive (opened eyes to sound but not closing them on request, moving head when asked but otherwise appeared vacant). Age 27. Repetitive high amplitude generalized sharp waves and spikes seen. D. Encephalopathic post-seizure. Patient partially responsive (turned head in response to name being called but otherwise vacant and unresponsive). Age 26. Diffuse high amplitude theta and delta activity, occasional multifocal sharp waves were noted. E. Also encephalopathic post-seizure. Patient drowsy but responding appropriately. Age 25. Diffuse theta and delta activity. Vertex phenomena also noted. Note differences between panel C and D; patient in an apparently similar clinical state but EEG in C shows continuous repetitive sharp waves/spikes. These are not present in panel D.
2.5 Plasma amino acids, urine amino acids, acylcarnitines and organic acids, cerebrospinal fluid examination for intermediary metabolites were normal as were neuronal antibodies and genetic testing with comparative genomic hybridisation array, karyotyping for ring chromosomes, mitochondrial disorders, and single gene tests (*BTD*, *SCN1A*). A whole exome sequencing panel (GEMINI, Cambridge University Hospitals NHS Foundation Trust) identified a heterozygous c.3312C > A, p.(Tyr1107Ter) truncating variant in *SMC1A*, which is absent from population databases. X-inactivation studies were not performed. Her parents were not available to assess *de novo* status.

NB – Clobazam used short term throughout.
2.4 Multiple magnetic resonance imaging (MRI) examinations of the brain since presentation at age 12 years have been normal with a notable lack of atrophy. Positron emission tomography-computed tomography at the age of 25 years showed mild, relatively diffuse, left cerebral hemisphere hypometabolism most evident in the frontal and parietal lobes. Echocardiography has shown a small ventricular septal defect at the apex of the left ventricle. Nerve conduction studies were consistent with a mild, likely axonal neuropathy. Electroencephalographic (EEG) examinations have demonstrated intermittent focal and multifocal epileptiform activity in various locations including the right anterior quadrant, the anterior hemispheres, and the parietal regions, alongside profound encephalopathy. Other typical EEGs showing generalised ictal changes are shown in Fig. 1.

## 3 Discussion

3.1 All previously reported cases of epilepsy due to truncating *SMC1A* variants describe a drug-resistant epilepsy with an onset in early childhood and moderate to severe intellectual disability, without the characteristic craniofacial dysmorphic features of CdLS and consistent with a DEE. This would be in keeping with the predominant mode of ascertainment of cases, the DDD project [3]. In this case, we hypothesize that the late age of seizure onset is concordant with the milder and later onset of neurocognitive compromise. Although similar to previously reported cases, she has a drug-resistant epilepsy with clustering of seizures and fever sensitivity, she does not display the characteristic dysmorphic features of CdLS. She has shown cognitive regression without atrophy detectable by MRI, temporally associated with frequent and intractable clusters of seizures/*status epilepticus*, in keeping with an epileptic encephalopathy [16].

This prominent clustering of seizures mirrors the clusters seen in *PCDH19-*related epilepsy, which similarly, is also characterised by fever-sensitive seizures, and a spectrum of cognitive features [17]. Whilst dysmorphic features are less common in *PCDH19-*related epilepsy and seizures are markedly fever sensitive, our case shows significant overlap with the *PCDH19* phenotype.

There is no evidence that any particular antiseizure medication is preferred for this group of patients. However, there has been reported benefit from ketogenic or the modified Atkins diets in three people reported in the literature [3], [13].

The patient described in this case developed an eighteen-month period of seizure freedom coinciding with the introduction of levonorgestrel/ethinylestradiol to treat a catamenial tendency. There is evidence that neurosteroids, particularly the progesterone metabolite allopregnanolone, are implicated in the pathophysiology of catamenial epilepsy. Allopregnanolone is a positive allosteric modulator of GABA_A_ neurotransmission and therefore withdrawal of progesterone, and subsequently allopregnanolone, during the menstrual cycle is thought to be related to the observed increase in seizure frequency [18]. The ketogenic diet has also been associated with altered neurosteroid metabolism, in particular neurosteroidogenesis and subsequent potentiation of GABA. This is thought to be a possible mechanism underpinning the use of the ketogenic diet in epilepsy [19]. Given the previous reports of improvement with a ketogenic or modified Atkins diet in three patients with *SMC1A* associated epilepsy and the initial improvement in our patient with levonorgestrel/ethinylestradiol we speculate that neurosteroid modulation may have a role in the treatment of *SMC1A* associated epilepsy.

The mechanism by which heterozygous truncating *SMC1A* variants cause the observed phenotype is unknown. The absence of reports documenting truncating *SMC1A* variants in men or boys, suggests that in these truncating variants may be incompatible with life — no predicted loss of function variants (such as protein truncating variants) are documented in gnomAD, indicating high constraint [20]. *SMC1A* is known to variably escape X-inactivation, with women shown to express twice as much *SMC1A* mRNA as men [21], [22]. This suggests that if *SMC1A* largely escapes X-inactivation in those with truncating *SMC1A* variants, haploinsufficiency is unlikely to be the cause of the observed phenotype as these women would have the equivalent expression of *SMC1A* to that of a normal male. Thus, mutant *SMC1A* may, instead, exert a dominant negative effect. Alternatively, there may be differences in biology between males and females that mean that lower levels of SMC1A can be better tolerated by women than men. However, if X-inactivation of truncating *SMC1A* variants does occur to some degree, this could lead to brain function in which there is significant cellular heterogeneity in SMC1A biology, with enough cells containing sufficient SMC1A to be viable, albeit predisposed to epilepsy and neurodevelopmental disorders. Conversely, the uniform loss of SMC1A function in male fetuses carrying hemizygous protein truncating variants may have more severe consequences that cannot be tolerated.

## 4 Conclusion

This case broadens the spectrum of *SMC1A* associated epilepsy in people without CdLS and with a DEE to include an adult female with normal neurodevelopment prior to seizures starting in late childhood. Truncating *SMC1A* variants may be considered as a potential cause of epilepsy with seizure clusters associated with drug-resistant epilepsy, and occur in adults with normal development prior to seizure onset.

## Declaration of Competing Interest

The authors declare the following financial interests/personal relationships which may be considered as potential competing interests: R.H.T. reports Honoraria from Arvelle, Bial, Eisai, GW Pharma, Sanofi, UCB Pharma, UNEEG and Zogenix. R.F., M.E., D.L.-S., A.W., and M.R.B. have nothing to declare.

## References

[1] Smith L., Singhal N., El Achkar C.M., Truglio G., Rosen Sheidley B., Sullivan J. (2018). *PCDH19* -related epilepsy is associated with a broad neurodevelopmental spectrum. Epilepsia.

[2] Vlaskamp D.R.M., Bassett A.S., Sullivan J.E., Robblee J., Sadleir L.G., Scheffer I.E. (2019). Schizophrenia is a later-onset feature of *PCDH19* Girls Clustering Epilepsy. Epilepsia.

[3] Symonds J.D., Joss S., Metcalfe K.A., Somarathi S., Cruden J., Devlin A.M. (2017). Heterozygous truncation mutations of the *SMC1A* gene cause a severe early onset epilepsy with cluster seizures in females: Detailed phenotyping of 10 new cases. Epilepsia.

[4] Musio A. (2020). The multiple facets of the SMC1A gene. Gene.

[5] Kline A.D., Moss J.F., Selicorni A., Bisgaard A.-M., Deardorff M.A., Gillett P.M. (2018). Diagnosis and management of Cornelia de Lange syndrome: first international consensus statement. Nat Rev Genet.

[6] Verrotti A., Agostinelli S., Prezioso G., Coppola G., Capovilla G., Romeo A. (2013). Epilepsy in patients with Cornelia de Lange syndrome: A clinical series. Seizure.

[7] Huisman S., Mulder P.A., Redeker E., Bader I., Bisgaard A.-M., Brooks A. (2017). Phenotypes and genotypes in individuals with *SMC1A* variants. Am J Med Genet A.

[8] Gorman K.M., Forman E., Conroy J., Allen N.M., Shahwan A., Lynch S.A. (2017). Novel SMC1A variant and epilepsy of infancy with migrating focal seizures: Expansion of the phenotype. Epilepsia.

[9] Goldstein J.H.R., Tim-aroon T., Shieh J., Merrill M., Deeb K.K., Zhang S. (2015). Novel SMC1A frameshift mutations in children with developmental delay and epilepsy. Eur J Med Genet.

[10] Jansen S., Kleefstra T., Willemsen M.H., de Vries P., Pfundt R., Hehir-Kwa J.Y. (2016). De novo loss-of-function mutations in X-linked SMC1A cause severe ID and therapy-resistant epilepsy in females: expanding the phenotypic spectrum. Clin Genet.

[11] Kruszka P., Berger S.I., Casa V., Dekker M.R., Gaesser J., Weiss K. (2019). Cohesin complex-associated holoprosencephaly. Brain.

[12] Lebrun N., Lebon S., Jeannet P.-Y., Jacquemont S., Billuart P., Bienvenu T. (2015). Early-onset encephalopathy with epilepsy associated with a novel splice site mutation in SMC1A. Am J Med Genet A.

[13] Naik N.A., Shah A.R. (2021). X linked Infantile Epileptic Encephalopathy due to SMC1A Truncating Mutation. Ann Indian Acad Neurol.

[14] Mioshi E., Dawson K., Mitchell J., Arnold R., Hodges J.R. (2006). The Addenbrooke’s Cognitive Examination Revised (ACE-R): a brief cognitive test battery for dementia screening. Int J Geriatr Psychiatry.

[15] Wechsler D. (2012). Wechsler Adult Intelligence Scale-Fourth Edition.

[16] Scheffer I.E., Liao J. (2020). Deciphering the concepts behind “Epileptic encephalopathy” and “Developmental and epileptic encephalopathy”. Eur J Paediatr Neurol.

[17] Oguni H., Nishikawa A., Sato Y., Otani Y., Ito S., Nagata S. (2019). A missense variant of SMC1A causes periodic pharmaco-resistant cluster seizures similar to PCDH19-related epilepsy. Epilepsy Res.

[18] Herzog A.G. (2015). Catamenial epilepsy: Update on prevalence, pathophysiology and treatment from the findings of the NIH Progesterone Treatment Trial. Seizure.

[19] Forte N., Medrihan L., Cappetti B., Baldelli P., Benfenati F. (2016). 2-Deoxy- d -glucose enhances tonic inhibition through the neurosteroid-mediated activation of extrasynaptic GABA _A_ receptors. Epilepsia.

[20] gnomAD n.d. https://gnomad.broadinstitute.org/(accessed September 15, 2021).

[21] Liu J., Feldman R., Zhang Z., Deardorff M.A., Haverfield E.V., Kaur M. (2009). SMC1A expression and mechanism of pathogenicity in probands with X-Linked Cornelia de Lange syndrome. Hum Mutat.

[22] Zhang Y., Castillo-Morales A., Jiang M., Zhu Y., Hu L., Urrutia A.O. (2013). Genes That Escape X-Inactivation in Humans Have High Intraspecific Variability in Expression, Are Associated with Mental Impairment but Are Not Slow Evolving. Mol Biol Evol.

